# The Clinical Investigation of Disparity of Utility Values Associated with Gallstone Disease: A Pilot Study

**DOI:** 10.1155/2013/216957

**Published:** 2013-09-11

**Authors:** Chung-Te Hsu, Yi Liao, Jorn-Hon Liu, Tao-Hsin Tung

**Affiliations:** ^1^Cheng Hsin General Hospital, Pai-Tou, Taipei 112, Taiwan; ^2^The First Affiliated Hospital of Wenzhou Medical College, Zhejiang, Wenzhou 325000, China; ^3^Faculty of Medicine, School of Medicine, National Yang-Ming University, Pai-Tou, Taipei 112, Taiwan; ^4^Faculty of Public Health, School of Medicine, Fu-Jen Catholic University, Xinzhuang, Taipei 24205, Taiwan

## Abstract

*Purpose*. The utility evaluation was an effective method to incorporate all of the contributing variables for multiple diseases into one outcome measure. A cross-sectional study was conducted to assess the utility values associated with varying states of gallstone disease among outpatient clinics participants at a teaching hospital in Taipei, Taiwan. *Methods*. The utility values were measured by using time trade-off method. A total of 120 outpatient clinics participants (30 subjects with no gallstone disease, 30 subjects with single stone, 30 subjects with multiple stones, and 30 subjects with cholecystectomy) evaluated utility values from January 1, 2006 to December 31, 2006. The diagnosis of gallstone disease was performed by a panel of specialists using ultrasound sonography. *Results*. The overall mean utility value was 0.89 ± 0.13 (95% CI: 0.87–0.91) indicating that study participants were willing to trade about 11% (95% CI: 9–13%) of their remaining life in return for being free of gallstone disease perpetually. The significant associated factors of utility values based on the multiple linear regression analysis were older age and different degrees of gallstone disease. *Conclusion*. Our results found that in addition to older age, multiple stones and cholecystectomy could influence utility values from the patient's preference-based viewpoint.

## 1. Introduction

Gallstone disease (GSD), a digestive disorder with multifactorial origins, was one of the major public health problems in the Western world [1, 2]. The economic and health impacts were also significantly related to its relatively higher morbidity. Of the 35% patients who developed the symptoms, around 80% experience biliary colic [3, 4]. Previous studies showed that the direct and indirect costs of treating GSD patients were estimated at $16 billion and account for more than 800,000 hospitalizations yearly in the United States [5, 6]. However, only a few studies have focused on the implications for maintenance of or improvement in the quality of life among GSD patients [7–11].

Contemporary clinical trials commonly measure end points such as quality of life and medical costs to establish whether therapies were both effective and cost effective [12]. From the preference-based viewpoint of the patient, the utility evaluation was an appropriate method to incorporate all of the contributing variables for multiple diseases into one outcome measure [12, 13]. Objectively, the measurement of utility values was a pattern that enabled evaluating quality of life [14]. In theory, a utility value of 1.0 was associated with full health, while a utility value of 0.0 was associated with death. The closer the utility value was to 1.0, the better a person could function in the activities of everyday life, whereas the closer the value to 0.0, the poorer the quality of life in health status [12–14].

Despite improved nutritional status and living standards in Taiwan, a steady increase was also reported in surgical treatment of GSD [15]. The natural history of GSD is usually benign but following complications contribute substantially to healthcare costs and may even be affecting quality of life although the progression of asymptomatic stage to symptomatic stage is relatively low, ranging from 10% to 25% [16, 17]. From the preventive medicine viewpoint, in order to reduce healthcare expenditures of GSD, organized preventive strategies were recommended. In Taiwan, there were few evidence-based medical studies concerning the utility values related to GSD. The purpose of this study was to assess utility values associated with varying states of GSD at a teaching hospital in Taipei, Taiwan.

## 2. Methods

### 2.1. Data Resource and Data Collection

This hospital-based cross-sectional study was conducted with a total of 120 outpatient clinic sites (OPD) participants aged 30 years or above (30 subjects with no gallstone disease, 30 subjects with single stone, 30 subjects with multiple stones, and 30 subjects with cholecystectomy) between January 1, 2006 and December 31, 2006. For the ethics consideration, subjects eligible for participation were first asked whether they would be willing to answer questions related to a utility survey and confirmed their willingness to participate by signing a consent form. People who were unwilling to answer the utility questions related to GSD were excluded from the study. In addition, access to hospital records was approved by the hospital human subjects review board at Cheng-Hsin General Hospital (CHGH-IRB: (165)98-26). All procedures were performed in accordance with the guidelines of our institutional ethics committee and adhered to the tenets of the Declaration of Helsinki. All subjects' information was anonymous.

The utility value, related demographic information (sex, age, education, and marriage status), and other personal chronic diseases history were collected at one-to-one well-trained interviews using a structured questionnaire. Biological factors (BMI, fasting plasma glucose, total cholesterol, triglyceride, aspartate aminotransferase (AST), and alanine aminotransferase (ALT)) were collected from fasting blood samples drawn by medical technologists.

### 2.2. Diagnosis of Gallstone Disease

In this study, GSD was diagnosed by a panel of specialists using real-time ultrasound sonography (TOSHIBA nemio SSA-550A, Japan) to examine the abdominal region after fasting for at least 8 hours based on the presence of movable hyperechoic material with acoustic shadow. Cases of GSD were classified as follows: single gallbladder stone, multiple gallbladder stones, and cholecystectomy, excluding gallbladder polyps [18].

In order to set up a consistent diagnosis of GSD between specialists, the weighted Kappa statistic was used to assess the agreement of interobserver reliability among study specialists. A pilot study was performed using 30 randomly selected subjects which included four states of GSD other than the study participants. For interobserver reliability, the weighted Kappa value for diagnosis of GSD between specialists was 0.77 (95% CI: 0.64–0.89).

### 2.3. Time Trade-Off Evaluation

The utility evaluation from the time trade-off (TTO) method in this study was used as a standard procedure [13, 19, 20]. The whole scenario was described as follows: “To suppose a situation that if you could live ten years under current health status. Now there is an opportunity that could return your health status to full health. This opportunity could increase your quality of life but decrease your survival. What is the maximum number of years you would be willing to give up if you could receive this opportunity and have full health for the remainder of your life?” The utility value was then calculated by dividing the number of years a subject was willing to trade in return for improving his or her life by the estimated number of years of remaining life and subtracting this number from 1.0, that is, utility value = 1.0 − (time traded/time of remaining life) [13]. For example, if a subject is expected to live ten additional years and would trade one year of them in return for full health status, the utility value would be calculated as 0.9 (1.0 − (1/10)). All information of utility values was collected by one well-trained interviewer in order to maintain consistency of interview quality.

### 2.4. Data Analysis

Statistical analysis was performed using SPSS 17.0 (SPSS, Chicago, IL). In the univariate analysis, the independent *t*-test method or one-way ANOVA was adopted to assess the differences of the mean value of utility. The multiple linear regression model was used to assess the independent effects of relevant factors on utility values after adjustment for the covariates. The information gathered from the study subjects was also evaluated by calculating appropriate standard deviations and 95% confidence intervals (CI). In addition, Bonferroni correction was performed to control multiple comparisons for multiple linear regression models.

## 3. Results

Table 1 presented the gender and age distribution of each state of GSD amongst study OPD participants. There were no statistically significant gender (*χ* ^2^-test = 0.64, *P* = 0.89) and age (*χ* ^2^-test = 7.42, *P* = 0.28) differences for each state of GSD.

Table 2 showed the results of distribution of utility values for study subjects. The distribution of utility values among study OPD participants had the statistically significant difference in each value category (*P* < 0.001). More than 40% of the participants answered that their utility value was 1.0, that is, had the full health status. Only about 8% study subjects answered that the utility values were below 0.7. Twelve (10%) participants were unable to answer how many years they would trade for full health when presented with the question. The remaining 108 study subjects were used for further analysis. Thus, the overall mean utility value was 0.89 ± 0.11 (95% CI: 0.87–0.91) and suggested that these subjects were willing to trade about 11% (95% CI: 9–13%) of their remaining life in return for being free of GSD perpetually. In addition, there was 49.1% of participants (53/108) who answered that the utility was 1.0 after excluding subjects who were unable to answer the utility value. That means only 50.9% (*n* = 55) of the subjects were willing to trade remaining year of life to be free from GSD and cholecystectomy. The mean utility value then was recalculated as 0.78 ± 0.10 (95% CI: 0.75–0.81) among the study subjects who were willing to trade year of life.

Table 3 showed the results of univariate analysis of utility values for study participants. The factors that were significantly related to utility values included age (≥65 yrs (0.85 ± 0.12, 95% CI: 0.81–0.89) versus <65 yrs (0.91 ± 0.13, 95% CI: 0.88–0.94), *P* = 0.02), different states of GSD (no GSD (0.93 ± 0.11, 95% CI: 0.89–0.97), single stone (0.92 ± 0.14, 95% CI: 0.88–0.97), multiple stones (0.88 ± 0.10, 95% CI: 0.84–0.92), and cholecystectomy (0.84 ± 0.14, 95% CI: 0.79–0.89), *P* = 0.02), and marriage (yes (0.91 ± 0.11, 95% CI: 0.88–0.94) versus no or widow (0.84 ± 0.15, 95% CI: 0.79–0.89), *P* = 0.02).

The effects of independent associated factors for the utility values after adjustment for confounding factors, that is, all univariate significant factors were examined with the multiple linear regression model using enter method.  Table 4 showed that older age and different states of GSD were the independent factors to affect the utility values after adjustment for confounders.

## 4. Discussion

### 4.1. The Clinical Implication of Utility Values

To understand disease-related quality of life and health status was a global issue for practice physicians. Outcomes data collected from various health care systems could enhance our understanding of disease impact and its treatment effectiveness [21]. Previous studies explored the quality of life associated with GSD patients by different measurement tools like SF-36, Psychological General Well Being Index (PGWB), and Nottingham Health Profile Part II (NHP), all had been extensively tested and validated [7, 8]. To evaluate utility values by TTO approach was not similar to most quality of life measurement tools that just focused on the task-specific orientation. Basically, the utility scores could quantify the subject's degree of impairment and function in the daily life by the disease status [13]. Utility values not only could be used to assess one's quality of life more comprehensively, but also could be compared to quality of life across widely different health status containing changeful medical specialties [13]. The theoretical ability of utility analysis to be more containing than other quality-of-life measures, as well as its capability to assess objectively the quality of life related to health states across all medical specialties [22].

The improvement in a utility value after a treatment could be used to evaluate the values of specific treatment for improving quality of life objectively [14]. Health outcome measures that combine length of life and health status into a single measure were useful in health planning and economic evaluation [20]. We could multiply the number of years of conferred treatment benefits to derive the number of quality-adjusted life years (QALYs) as medical treatments could improve the subject's utility value [13, 23]. For example, as Table 3 showed, cholecystectomy could treat GSD, that is, the utility value of study patients with GSD was increased from 0.84 to 0.93, there will be a 0.09 increase from the therapy. The number of QALYs from the cholecystectomy therapy then would be obtained as 1.8 (0.09 × 20) if those subjects had a life expectancy of 20 years.

In this study, approximately 45% of surveyed subjects reported perfect health status with a utility value of 1.0. It implied that unless they experienced cholecystectomy, only GSD syndrome could not seriously affect the activities of daily living and functional quality to these subjects. Previous population-based studies showed that the proportion of respondents not trading of any time when answering the TTO question (TTO value of 1.0) was more than 50% [13, 20]. The proportion of respondents not trading of any time in this study was 44.2%. The hospital-based study design might cause the potential selection bias. Another possible reason was that TTO method was unlikely to be sensitive to the direct measurement of preferences for all but the worst temporary health states when the scale required a direct trade off between the temporary health state and the quantity of life [19].

### 4.2. Clinical Factors Associated with the Utility Values

It was not surprising that the aged people had lower utility values than young ones in this. Other quality of life studies also demonstrated the same findings [17]. In Taiwan, although the nutritional status and living standard had improved, there is a steady increase in surgical treatment of GSD during the past four decades [15, 17]. The trend of ageing also implied that elderly people usually represented the worse healthcare groups, with less social and family support and would need more medical resources [13]. The improvement of GSD substantiality by appropriate prevention and treatment might ameliorate quality of life related to the disease. In addition, previous study showed that some serum biochemical levels significantly predict the quality of life and could be used to evaluate patient's well-being at admission [17]. Neither gender, level of education, marriage status, other chronic diseases, BMI, nor some serum biochemical results were significantly and independently affecting the utility values. Further epidemiological and etiologic investigations were needed to clarify the pathophysiological mechanisms between related factors and utility values among GSD populations.

The utility values by TTO method showed statistically significant increase in subjects with no GSD than those who are with GSD. The results in this study implied that from patient's preference-based viewpoint, subjects with better state of GSD owned better quality of life than those who were in the worse state. In addition, one would anticipate that following the short term postoperative recovery that patients with cholecystectomy would then not have further pain and would have returned to their premorbid state with a better quality of life. The overall improvement in quality of life also has been demonstrated after cholecystectomy in the previous studies [7–9]. In this study, however, the subgroup of “patients with cholecystectomy” means they are assessing immediate postoperative condition reflecting postoperative pain. This might explain why the utility values of patients with cholecystectomy in this study were significantly different to patients with no GSD. The utility value of study subjects with cholecystectomy was 0.84, it not only revealed the cholecystectomy group was willing to trade more of their remaining life years (16%, 95% CI: 11%–21%) than other GSD groups, but also suggested the importance of gastrointestinal function maintenance. The appropriate integrated diagnosis and therapy in the early stage of GSD could assure the quality of life among this population.

### 4.3. Study Population and Methodological Consideration

There were some strengths of methodology in this study. From the viewpoint of evidence-based medicine, utility values could be applied for further economic evaluation. The utility measurement could be viewed as the key in deriving objective results from economic evaluation that takes into consideration maintenance or improvement in quality of life and length of year [13]. We could obtain QALYs in this study population of interest over a period of time. Because of the importance of the effect on QALYs of each treatment, a cost utility analysis (CUA) was selected as the appropriate framework for an economic evaluation. CUA evaluates treatment outcomes, not only in terms of the quantity of life obtained, but also in terms of the “quality of life” gained from the intervention for GSD patients [24, 25]. In addition, we have adjusted other possible nondisease health-related factors which might confound the utility values by using linear regression model.

Nevertheless, this study still had an inevitable weakness which is repeated questions to measure the utility and which previous studies also mentioned [13, 22]. Our measurements were done only at a single point in time and would not be able to be used to reflect long-term exposure to various demographic or biochemical aspects or factors, factors which might be important influencers of utility values. The rating of such an option would be so seriously distorted by the imminence of death that it is unlikely that answers would bear any relation to the utility of the health state revealed in another context [16]. The solution to such a quandary is to conduct a number of prospective longitudinal analogous studies, the results of which would be expected to complement the cross-sectional findings of this study. We also did not consider how many individuals had progressive liver disease and did not explore the relationship between GSD and liver disease. Quota sampling method is used in this study due to the fact that sampling frame is not available. However, this sampling method is the nonprobability version of stratified sampling. The problem of quota sampling is that these samples may be biased because not everyone has a chance of selection. This nonrandom element is a source of uncertainty about the nature of the actual sample and quota. In addition, the potential selection bias due to the hospital-based study design, that is, of it not being exactly representative of the whole general population. Furthermore, the subjects with cholecystectomies had lower utility values compared to the groups with no, single, and multiple gallstones. The information was limited if this group was compared with asymptomatic subjects with cholelithiasis because most likely symptoms or complications of GSD led to the operation. Finally, a lower sample size in this study was another drawback. We consider the results of only a pilot study due to its really hard to gain, strong confidence intervals for mean utilities with this size sample. Further epidemiological and follow-up investigations of similarly defined groups with larger study sample sizes were needed to clarify the results more precisely and temporality between degrees of GSD and utility values.

## 5. Conclusion

In conclusion, we have used TTO method to quantify the utility values of patients with or without GSD. Our results found that in addition to older age, multiple stones and cholecystectomy could influence utility values from the patient's preference-based viewpoint.

## Figures and Tables

**Table 1:** The gender and age distribution of the utility survey among outpatient clinics participants (*n* = 120).

Variable	No gallstone disease		Single stone		Multiple stone		Cholecystectomy		Total		*P* value for *χ* ^2^-test
	*n*	%	*n*	%	*n*	%	*n*	%	*n*	%	
Sex											
Male	14	46.7	12	40.0	15	50.0	14	46.7	55	45.8	0.89
Female	16	53.3	18	60.0	15	50.0	16	53.3	65	54.2	
Age											
30–49	9	30.0	8	26.7	5	16.7	4	13.3	26	21.7	0.28
50–64	14	46.7	16	53.3	15	50.0	12	40.0	57	47.5	
65+	7	23.3	6	20.0	10	33.3	14	46.7	37	30.8	

Total	30	100.0	30	100.0	30	100.0	30	100.0	120	100.0	

**Table 2:** The distribution of utility values by time trade-off method for gallstone disease (*n* = 120).

Utility values	Number	(%)	*χ* ^2^-test for equal proportion
1.0	53	44.2	
0.9–1.0	14	11.6	
0.8–0.9	17	14.2	*P* < 0.001
0.7–0.8	14	11.7	
<0.7	10	8.3	
Unable to answer	12	10.0	
Total	120	100.0	
Mean ± SD* (*n* = 108) (95% CI)	0.89 ± 0.13 (0.87–0.91)		
Mean ± SD** (*n* = 55) (95% CI)	0.78 ± 0.10 (0.75–0.81)		

*Excluded participants who were unable to answer how many years they would trade for being rid of gallstone disease.

**Only calculated subjects who were willing to trade time of their life years to be rid of gallstone disease and cholecystectomy.

**Table 3:** Univariate analysis of utility values for outpatient clinics participants (*n* = 108).

Variable	No	(%)	Utility value		*P* value for *t*-test or ANOVA
			Mean ± SD	95% CI	
Sex					
Male	48	44.4	0.88 ± 0.12	0.85–0.91	0.51
Female	60	55.6	0.90 ± 0.14	0.86–0.94	
Age					
<65 yrs	75	69.4	0.91 ± 0.13	0.88–0.94	0.02
≥65 yrs	33	30.6	0.85 ± 0.12	0.81–0.89	
Gallstone disease (GSD)					
No GSD	26	24.1	0.93 ± 0.11	0.89–0.97	0.02
Single stone	27	25.0	0.92 ± 0.14	0.88–0.97	
Multiple stones	27	25.0	0.88 ± 0.10	0.84–0.92	
Cholecystectomy	28	25.9	0.84 ± 0.14	0.79–0.89	
Education					
Senior high school or above	63	58.3	0.90 ± 0.12	0.87–0.93	0.45
Junior high school or below	45	41.7	0.88 ± 0.14	0.84–0.92	
Marriage					
Yes	72	66.7	0.91 ± 0.11	0.88–0.94	0.02
No or widow	36	33.3	0.84 ± 0.15	0.79–0.89	
BMI					
<25 Kg/m^2^	43	39.8	0.90 ± 0.14	0.86–0.94	0.48
≥25 Kg/m^2^	65	60.2	0.88 ± 0.12	0.85–0.91	
Total cholesterol					
<200 mg/dL	64	59.3	0.90 ± 0.14	0.87–0.94	0.19
≥200 mg/dL	44	40.7	0.87 ± 0.12	0.83–0.91	
Triglyceride					
<200 mg/dL	70	64.8	0.90 ± 0.13	0.87–0.93	0.20
≥200 mg/dL	38	35.2	0.87 ± 0.12	0.83–0.91	
Fasting plasma glucose					
<126 mg/dL	68	63.0	0.90 ± 0.13	0.87–0.93	0.35
≥126 mg/dL	40	37.0	0.88 ± 0.13	0.84–0.92	
AST					
<40 U/L	97	90.0	0.89 ± 0.13	0.86–0.92	0.64
≥40 U/L	11	10.0	0.87 ± 0.15	0.78–0.96	
ALT					
<40 U/L	95	88.0	0.89 ± 0.13	0.86–0.92	0.95
≥40 U/L	13	12.0	0.89 ± 0.14	0.81–0.97	
Other chronic diseases					
No	69	63.4	0.90 ± 0.13	0.87–0.93	0.26
Yes	39	36.6	0.87 ± 0.13	0.83–0.91	

**Table 4:** Multiple linear regression on the associated factors related to the utility values that all univariate significant factors were included among outpatient clinics participants (*n* = 108).

Variables	*β*	SE	95% CI	*P* value
Intercept	0.927	0.048	0.823~1.023	<0.001
Sex (male versus female)	0.005	0.043	−0.081~0.091	0.91
Age (≥65 versus <65 yrs)	−0.103	0.042	−0.187~−0.020	0.02
Gallstone disease (GSD)				
Single stone versus no GSD	−0.036	0.057	−0.148~0.077	0.53
Multiple stones versus no GSD	−0.140	0.055	−0.248~−0.031	0.01
Cholecystectomy versus no GSD	−0.258	0.056	−0.368~−0.147	<0.001
Marriage (yes versus no + widow)	0.048	0.042	−0.035~0.132	0.25

The significant level of *P* value was 0.016 (0.05/3) for Bonferroni correction to multiple comparisons among single stone versus no GSD, multiple stones versus no GSD, and cholecystectomy versus no GSD.
